# Estimation of the benchmark dose of urinary cadmium as the reference level for renal dysfunction: a large sample study in five cadmium polluted areas in China

**DOI:** 10.1186/s12889-015-2021-x

**Published:** 2015-07-14

**Authors:** Shen Ke, Xi-Yu Cheng, Jie-Ying Zhang, Wen-Jing Jia, Hao Li, Hui-Fang Luo, Peng-He Ge, Ze-Min Liu, Hong-Mei Wang, Jin-Sheng He, Zhi-Nan Chen

**Affiliations:** College of Life Sciences and Bioengineering, School of Science, Beijing Jiaotong University, Beijing, 100044 People’s Republic of China; State Key Laboratory of Environmental Criteria and Risk Assessment, Chinese Research Academy of Environmental Sciences, Beijing, 100012 People’s Republic of China

**Keywords:** Urinary cadmium, Benchmark dose, Renal dysfunction, Urinary β_2_-microglobulin, Large sample population

## Abstract

**Background:**

Itai-itai disease primarily results from cadmium (Cd) exposure and is known as one of the four major pollution diseases in Japan. Cd pollution is more serious in several areas of China than in Japan. However, there is still a lack of information regarding the threshold level of Cd exposure for the adverse health effects in the general Chinese population. This study aims to evaluate the reference value of urinary Cd (UCd) for renal dysfunction in a Chinese population as the benchmark dose lower confidence limit (BMDL) based on a large sample survey.

**Methods:**

A total of 6103 participants who lived in five Cd polluted areas of China participated in this study. We analyzed UCd levels as a biomarker of exposure and urinary β_2_-microglobulin (Uβ_2_-MG) levels as a renal tubular effect biomarker. The BMD studies were performed using BMD software. The benchmark response (BMR) was defined as a 10 % additional risk above the background.

**Results:**

There was a positive correlation between the UCd levels and the prevalence of Uβ_2_-MG. The BMD of UCd for Uβ_2_-MG was estimated for each province. The findings showed that the BMD levels were related to the participants’ geographic region, which may be partially due to the large differences in Cd exposure level, ethnic group, lifestyle and diet of the sample population in these study areas. The reference level of UCd for the renal effects was further evaluated by combining the five sets of data from all 6103 subjects. The overall BMDLs of UCd for Uβ_2_-MG with an excess risk of 10 % were 2.00 μg/g creatinine (μg/g cr) in males and 1.69 μg/g cr in females, which were significantly lower than the World Health Organization (WHO) threshold level of 5 μg/g cr for Cd-related renal effects.

**Conclusions:**

The selection of the sample population and geographic region affected the BMDL evaluation. Based on the findings of this survey of a large sample population, the UCd BMDLs for Uβ_2_-MG in males with BMRs at 10 % were 2.00 μg/g cr. The BMD was slightly lower in females, which indicated that females may be relatively more sensitive to Cd exposure than males.

## Background

Cadmium (Cd) is a non-essential element in the human body, and long-term exposure to sufficiently high Cd levels through food and water consumption, skin contact and inhalation may cause adverse health effects, such as renal dysfunction and osteoporosis [1–4]. Cd and its compounds are considered to be human carcinogens (primarily cancer resulting from inhalation exposures) [5], and Cd exposure is associated with breast cancer development in females [2]. Cd and its compounds are released to all elements of the environment due to a number of human activities, such as mining, smelting, industrial activities, waste disposal, application of fertilizer and pesticides and vehicle exhaust [6–8]. Cd pollution in soil, water and air, as well as different foods, has been frequently reported in China in recent years, which imposes increasing health risks to the public [8, 9]. To protect people from the adverse effects of Cd exposure, it is important to evaluate the reference level, which is the lowest concentration of urinary Cd (UCd) with a low probability of Cd-induced adverse health effects.

The benchmark dose (BMD) represents an estimate of the dose corresponding to a specified level of increased response (the benchmark response, BMR). It has been suggested that the no observed adverse effect level (NOAEL) can be replaced with the lower 95 % confidence limit of BMD [10, 11]. Compared with the NOAEL, the BMD method is not constrained as one of the experimental doses and makes better use of the dose–response information. This method more appropriately reflects the sample size and is regarded as a better approach than NOAEL to estimate the reference point for a continuous outcome variable [12].

Previous studies have examined the reference level for Cd-induced kidney effects using the BMD method and have currently displayed relatively large variations in critical UCd levels [13–22]. The Japanese population data from previous studies suggested that the benchmark dose lower confidence limit (BMDL) of UCd for renal effects ranged from 2.5 μg/g cr to 10.3 μg/g cr in males and 1.4 μg/g cr to 11.4 μg/g cr in females [13–16]. Another study reported that the BMDLs of UCd were between 0.5 μg/g cr to 1.2 μg/g cr in a Swedish population [17], whereas some other researchers reported BMDLs ranging from 0.44 μg/g cr to 12.18 μg/g cr in Chinese populations [18–21]. The BMDL values may have been significantly affected by many factors, such as the sample population and Cd exposure levels, thereby necessitating further studies to identify the possible factors affecting the BMD evaluation.

Itai-Itai disease primarily results from cadmium (Cd) exposure and is known as one of the four major pollution diseases of Japan. Considering that Cd pollution is more serious in certain areas of China than in Japan, environmental epidemiological studies on Cd pollution in China clearly warrant more attention. However, there is still a lack of information on the reference level of Cd exposure for the adverse health effects in the general Chinese population. The limited studies on the Chinese population used a much smaller sample size than the sample sizes of the Japanese population reported in other studies (374 to 790 participants vs. 1270 to 3103 participants) [14–17, 19–21].

In the present study, a large sample survey of 6103 participants from five Cd-polluted provinces in China was performed to identify the reference level of UCd for renal dysfunction, with urinary β_2_-microglobulin (Uβ_2_-MG) serving as the renal effect biomarker. The BMD values of UCd for renal dysfunction in the population of each study province were evaluated individually to study the effect of gender and the sample population on the BMD. Furthermore, the overall BMD of UCd for renal dysfunction in the Chinese population was assessed by combining the five data sets from all 6103 subjects.

## Methods

### Area and study population

Industrial operations, particularly smelting and mining, are considered to be one of the most important sources of Cd pollution. Considering that the differences in the Cd exposure levels, lifestyle, and eating habits of the study population may be related to Cd’s toxic effects, we included population groups from different geographic regions (Hubei in central China, Guangdong in southern China, Gansu in northwest China, and Yunnan and Guizhou in southwest China) with known industrial Cd pollution, rather than from a single region. The five regions included one slightly exposed area (Hubei), and four moderately/highly exposed areas (Guangdong, Yunnan, Gansu and Guizhou). The Hubei province in central China had less industrial Cd contamination than other areas, with soil Cd concentrations ranging from 0.8 mg/kg to 1.47 mg/kg [23]. Extensive smelting and mining contributed to serious Cd pollution in the Guangdong, Yunnan, Gansu and Guizhou provinces. Dabaoshan Mine, located in Shaoguan, in the Guangdong province, is the largest polymetallic mine in southern China. Many mineral slag and acid mine effluents have been discharged into the local rivers from the beginning of mining operations in the 1970s. Gejiu in the Yunnan province, which is a well-known “Tin-producing city” in southwest China, has more than 400 nonferrous metal processing companies. Baiyin in the Gansu province, which is a well-known “Copper-producing city” in northwest China, has mined and smelted nonferrous metals (e.g., Cu, Pb and Zn) since the 1960s. In Hezhang in the Guizhou province in southwest China, an indigenous zinc-smelting method, which resulted in severe heavy metal pollution, has been used for more than a century [24, 25]. Mineral slag and mining and smelting wastewater have caused serious pollution of the soil and local rivers (e.g., the Kafangdagou River in Gejiu, Yunan province, the Dongdagou River in Baiyin, Gansu province and the Magu River in Hezhang, Guizhou province). Although low soil Cd contamination was also observed in some unpolluted regions in these provinces, the average soil Cd concentrations in some polluted areas were as high as 5.7 mg/kg to 10 mg/kg [7, 9, 24]. The Cd contamination of rice in the study areas described above has been monitored, and more than 18 % of those samples had Cd concentrations greater than 0.2 mg/kg, which is the current Chinese maximum allowable concentration (MAC) of Cd in rice (detailed information will be reported in another study).

Our study population included inhabitants of these five Cd polluted areas. Approximately 12,000 subjects were randomly selected from the towns and/or villages in these areas based on the information from local residents. They were invited to participate in this study by phone or by post. Individuals who responded positively (approximately 80 % of 12,000 subjects) were then asked to fill out a brief questionnaire. Subjects who had been diagnosed with kidney and/or liver disease were excluded from the study. Participants who were occupationally exposed to heavy metals were also excluded. The eligible subjects were asked to provide urine samples for biological measurements. A total of 6103 participants (51 % of 12,000 subjects), including 1496 (Gansu), 1565 (Hubei), 1239 (Guizhou), 736 (Guangdong), and 1067 (Yunnan), completed the questionnaires and provided urine samples. Of those who provided urine samples, 44 % (2715) were males and 56 % (3388) were females, age 35 and older. Women were slightly more likely than men to participate in this study and provide a urine sample. All eligible subjects drank water from the area at least once a week. Their primary occupation was farming. Table 1 presents the data on the number and ages of all participants from each province. Ethical permission was obtained from the Ethics Committee at Beijing Jiaotong University. Informed consent was obtained from all participants.

**Table 1:** Characteristics of the population used in the study

Area	Sex	No.	Age (mean, range)	UCd (μg/g cr) (GM, range)	Uβ_2_-MG (μg/g cr) (GM, range)	UCd > 5 (%)^a^	Uβ_2_-MG > 1000 (%)^a^	UCd > 5 & Uβ_2_-MG > 1000 (%)^a^
Gansu	Male	663	59.23 (35–87)	4.44 ± 2.47 (0.10 ~ 36.79)	660.87 (5.01–2911.50)	47.7	29.6	21.9
Hubei		724	60.45 (35–89)	4.69 ± 1.97 (0.21 ~ 33.32)	724.27 (2.42–5087.62)	46.4	38.8	23.8
Guizhou		548	59.26 (36–89)	6.60 ± 2.18 (0.08 ~ 56.99)	310.25 (6.50–4603.32)	67.7	19.5	17.0
Guangdong		321	62.39 (35–89)	4.06 ± 1.93 (0.81 ~ 24.86)	435.90 (0.76–2554.98)	33.6	29.3	11.8
Yunnan		459	61.13 (35–89)	4.37 ± 2.08 (0.56 ~ 31.96)	793.84 (4.91–5272.68)	36.4	42.7	20.5
Gansu	Female	833	60.42 (35–89)	4.34 ± 2.64 (0.11 ~ 48.47)	654.79 (14.92–3320.68)	47.8	28.8	20.2
Hubei		841	60.72 (35–89)	4.84 ± 2.02 (0.17 ~ 42.05)	727.56 (5.59–4527.47)	49.9	40.3	27.5
Guizhou		691	58.53 (35–89)	6.50 ± 2.26 (0.05 ~ 57.27)	348.03 (3.46–5926.02)	67.9	30.7	25.6
Guangdong		415	61.17 (35–89)	3.94 ± 2.12 (0.13 ~ 45.62)	474.92 (1.55–3216.06)	36.6	31.6	18.3
Yunnan		608	56.38(35–88)	4.76 ± 2.25 (0.07 ~ 47.90)	745.64 (14.97–5645.35)	44.6	41.3	24.8
All regions	Male	2715	60.26 (35–89)	4.82 (0.08–56.99)	570.86 (0.76–5272.68)	47.8	32.2	20.0
	Female	3388	59.48 (35–89)	4.87 (0.05–57.27)	581.46 (1.55–5926.02)	50.5	34.6	23.7

Notes: *GM* geometric mean.^**a**^The cut-off values for UCd and Uβ_2_-MG are expressed as μg/g cr

### Sampling and chemical analysis

Morning urine samples were collected from the eligible participants. Each sample was divided into several parts immediately after collection to measure the UCd, Uβ_2_-MG and urinary creatinine (cr) concentrations. UCd was determined using the standard method described in previous studies [19, 26]. In brief, the urine samples were collected in acid-washed plastic containers and frozen at −20 °C until further analysis. The urine samples were acidified with concentrated nitric acid, and the Cd concentrations in the urine samples were then determined by graphite-furnace atomic absorption spectrometry with peak area evaluation. The linearity range of the calibration curve (R^2^ = 0.996 to 0.999) was from 0 to 20 μg/L. The method was validated by evaluating the quality control sample and recovery. Two concentrations of the quality control samples (5.1 μg/L and 15.0 μg/L) were analyzed, which produced Cd concentrations of 4.9 ± 0.2 μg/L and 14.6 ± 0.4 μg/L. The relative standard deviations for Cd were 4.5 % and 2.7 % in the two quality control samples, respectively. The average recovery of adding different amounts of Cd for analyzing UCd was between 97.8 % and 103.0 %. The UCd limit of detection was 0.07 μg/L. For analytical quality assurance, one run of both the quality control samples and the calibration standards were used for every analytical run. Urinary β_2_-MG (Uβ_2_-MG) levels were measured by radio-immunoassay (RIA) (Pharmacia β_2_-micro RIA, Pharmacia Diagnostics AB, Sweden). Urinary creatinine (cr) levels were measured as previously described [19, 27]. All urinary parameters were adjusted for the creatinine concentration and expressed as μg/g creatinine (μg/g cr) [28].

### Statistical analysis

SPSS 17.0 for Windows (SPSS Inc., Chicago, IL, USA) and Origin 8.0 were used to conduct the statistical analyses. The variables were log-transformed to meet the normal distribution requirements. The age, UCd and Uβ_2_-MG means were calculated. The prevalence of the elevated UCd and Uβ_2_-MG levels was calculated, and the cut-off values were chosen according to available risk assessment–based biomarker screening values. The threshold values for UCd and Uβ_2_-MG used in this study are 5 μg/g cr [29] and 1000 μg/g cr [30, 31], respectively. One-way ANOVA was used to test for univariate differences between groups. The results were considered statistically significant at *p* <0.05 or 0.01 based on a two-tailed test. The data were expressed as the mean or geometric mean (GM).

### Benchmark dose (BMD) method

BMD studies were performed using BMDS (Version 2.3.1, Environmental Protection Agency, USA, http://www.epa.gov/ncea/bmds/dwnldu.html). The subjects were divided into four groups (UCd: 0–2, 2–6, 6–12 and >12 μg/g cr). On one hand, these subgroups were selected based on previous studies [13, 14, 18, 19] and the range and distribution of Cd exposure levels of the sample population in this study. On the other hand, having studies with one or more doses near the BMR level is desirable to give a better estimate of the BMD based on the Benchmark Dose Technical Guidance. The Uβ_2_-MG level of 1000 μg/g cr was used as the cut-off value [30, 31]. Based on the Benchmark Dose Technical Guidance, most fitting methods will provide a global goodness-of-fit measure, usually a *p*-value. These measures quantify the degree to which the dose-group means that are predicted by the model differ from the actual dose-group mean, relative to the amount of expected variation in the dose-group means. Small *p*-values indicate that the goodness-of-fit statistical value at least this extreme is unlikely to have been achieved if the data were actually sampled from the model, and, consequently, the model is a poor fit to the data. When the *p* values were higher than 0.05, the results of model fit were considered to be satisfactory [19]. In a preliminary study of this work, we tested different models, including the Gamma, Logistic, Log-Logistic, Log-Probit and others. Some of them have *p* < 0.05 for several study areas, and these were ruled out for further consideration. Among the remaining models, both the Gamma model and Log-logistic model fit the data well, and they had similar low Akaike’s Information Criterion (AIC) values (Based on the Benchmark Dose Technical Guidance, the models with low AIC values are preferred). Therefore, in most cases, the BMDs and BMDLs for Cd-induced renal dysfunction were calculated using a Gamma model based on the populations from the five provinces. When the equation was not a good fit, a Log-logistic model was chosen, as specified in the text. The BMR was defined as a 10 % additional risk above the background. The criterion significance level was set at *p* < 0.05.

## Results

### Internal Cd exposure and the prevalence of renal dysfunction in the subjects

A total of 6103 participants (2715 males and 3388 females) from five Chinese provinces were included in this survey. The subjects’ characteristics are shown in Table 1. The number of subjects in each province varied from 321 to 724 for males and 415 to 841 for females. These subjects were 35 years old or older, with the average ages ranging from 56.4 to 62.4. A wide range of UCd concentrations was observed (Table 1). The mean Cd levels of the subjects from the Guangdong province were statistically lower than the levels of the subjects from the other provinces (*p* < 0.05). The highest UCd level of 6.50-6.60 μg/g cr was recorded in the subjects from the Guizhou province. The threshold value of 5 μg/g cr that was set by the World Health Organization (WHO) [29] was used as the cut-off value to calculate the prevalence of the elevated UCd levels. Table 1 shows 67.7 % of males and 67.9 % of females of the sample population from the Guizhou province had measured UCd concentrations greater than the threshold value of 5 μg/g cr, as opposed to 33.6 %-49.9 % observed in the sample population from the other four study areas.

The mean Uβ_2_-MG levels of the subjects from the Guizhou and Guangdong provinces were statistically lower (*p* < 0.05), whereas the highest levels of 793.84 μg/g cr and 745.64 μg/g cr were observed in males and females, respectively, from the Yunnan province (Table 1). For all subjects of the five provinces, the mean Uβ_2_-MG levels were 570.86 μg/g cr in males and 581.46 μg/g cr in females. A Uβ_2_-MG reference point of 1000 μg/g cr for renal dysfunction was suggested by the Minister of Health of the People's Republic of China (MOHC) in 1998 [31]. It should be noted that the Uβ_2_-MG levels of some subjects significantly exceeded the safety limit, indicated by the 95th percentiles of the Uβ_2_-MG concentrations (1664.5-2689.8 μg/g cr, data not shown in the Tables). However, the mean Uβ_2_-MG levels of the subjects were statistically lower than the standard of 1000 μg/g cr (Table 1). The threshold value of 1000 μg/g cr was used as the cut-off value to calculate the prevalence of the elevated Uβ_2_-MG levels, and the results are shown in Table 1. In the sample population in the Guizhou province, 19.5 % of males and 30.7 % of females had measured UCd concentrations greater than the threshold value, whereas this value was exceeded in 29.3 %-42.7 % of males and 28.8 %-41.3 % of females in the sample populations in the other four study areas. Notably, both the UCd and Uβ_2_-MG levels exceeded the corresponding threshold levels in only 11.8 % of males and 18.3 % of females in the Guangdong province compared to 17.0 %–23.8 % of males and 20.2 %–27.5 % of females from the other study areas.

The subjects from each province were divided into four groups (UCd: 0 to 2, 2 to 6, 6 to 12 and >12 μg/g cr), and the frequency that exceeded the Uβ_2_-MG standard were then calculated accordingly. The results are shown in Table 2. The frequency that exceeded the Uβ_2_-MG standard ranged from 8.3 % to 36.4 % and from 12.8 % to 47.8 % for the male subjects from the Guizhou and Guangdong provinces, respectively. Compared to these subjects, a relatively higher frequency exceeded the Uβ_2_-MG standard was observed in the subjects from the other regions. For all subjects from the five provinces, the frequency exceeding the Uβ_2_-MG standard was 14.6 %–58.7 % in males and 14.5 %–65.4 % in females. It is clear that the frequency that exceeded the threshold level of Uβ_2_-MG increased with increasing UCd levels. The frequency that exceeded the threshold level of Uβ_2_-MG was within 14.6 % in all subjects when the UCd levels ranged from 0 μg/g cr to 2 μg/g cr. When the UCd levels were above 2 μg/g cr, a higher frequency was recorded (26.3–65.4 %). The Chi-squared linear trend test (*p* < 0.05) further verified that the prevalence of renal dysfunction was correlated with increased UCd levels. A dose–response relationship was apparent between the UCd levels and the prevalence of renal dysfunction, which is consistent with the findings of a previous study [18].

**Table 2:** The prevalence of renal dysfunction at different UCd levels in males and females living in the five Cd polluted areas in China

U-Cd (μg/g cr)	Uβ_2_-MG (Male)																	
	Gansu			Hubei			Guizhou			Guangdong			Yunnan			All regions		
	+	-	%	+	-	%	+	-	%	+	-	%	+	-	%	+	-	%
0-2	9	97	8.5	13	53	19.7	3	33	8.3	5	34	12.8	13	35	27.1	43	252	14.6
2-6	51	241	17.5	117	275	29.8	22	168	11.6	57	136	29.5	113	191	37.2	360	1011	26.3
6-12	83	112	42.6	127	104	55.0	43	172	20.0	21	45	31.8	29	27	51.8	303	460	39.7
12-	53	17	75.7	24	11	68.6	39	68	36.4	11	12	47.8	41	10	80.4	168	118	58.7
*χ*2	120.490			24.990			18.052			3.967			13.592			173.238		
*p*	2.2e–16			2.2e–14			1.8e–07			0.046			7.9e–10			2.2e–16		
U-Cd (μg/g cr)	Uβ_2_-MG (Female)																	
	Gansu			Hubei			Guizhou			Guangdong			Yunnan			All regions		
	+	-	%	+	-	%	+	-	%	+	-	%	+	-	%	+	-	%
0-2	20	140	12.5	10	68	12.8	0	41	0.0	13	55	19.1	17	49	25.8	60	353	14.5
2-6	66	280	19.1	135	301	31.0	52	200	20.6	50	173	22.4	115	231	33.2	418	1185	36.1
6-12	98	136	42.1	157	121	56.5	81	181	30.9	47	50	48.5	63	58	52.1	446	545	45.0
12-	56	38	59.6	37	12	75.5	79	57	58.1	21	6	77.8	56	19	74.7	249	132	65.4
*χ*2	93.391			94.632			72.993			44.668			53.056			324.985		
*p*	2.2e-16			2.2e-16			2.2e-16			2.2e-11			3.1e-13			2.2e-16		

Notes: Chi-squared linear trend test, *p* < 0.05

### The BMD of UCd for the renal dysfunction index in the population from each province

Next, the BMDL of UCd for Uβ_2_-MG was calculated for the population from each province using the BMD software from the Environmental Protection Agency (Version 2.3.1). The results for males are shown in Table 3. All *p* values were higher than 0.05, which indicates that the results of the model fit were satisfactory [19]. Using the Uβ_2_-MG as an indicator of renal dysfunction, the estimated BMDL values of UCd were between 1.10 μg/g cr and 2.11 μg/g cr in males from all provinces except the Guizhou province. At a BMR of 10 %, the BMDL of UCd in males from the Guizhou province reached 3.66 μg/g cr, which was appreciably higher than that of the males from the other provinces.

**Table 3:** The BMDL estimates of the UCd for Uβ_2_-MG in the different regions and all regions

Area	Sex	AIC	Chi-squared	*p*-value	BMD_10_ (μg/g cr)	BMDL_10_ (μg/g cr)
Gansu	Male	682.0	0.23	0.63	3.32	2.11
Hubei		914.0	3.21	0.07	2.01	1.13
Guizhou		518.5	0.13	0.72	6.56	3.66
Guangdong		386.2	3.4	0.18	3.66	2.02
Yunnan		591.3	0.04	0.98	2.33	1.10
All areas		3241.6	0.96	0.62	2.28	2.00
Gansu	Female	911.1	3.41	0.06	2.95	1.59*
Hubei		1042.6	4.28	0.12	1.16	1.00
Guizhou		774.6	3.58	0.17	2.25	1.94
Guangdong		474.8	2.15	0.14	3.20	1.37
Yunnan		774.8	1.08	0.30	2.23	1.33
All areas		4046.0	4.33	0.12	1.87	1.69

Notes: Confidence Level: 95 %, BMR: 10 %. ^a^The BMDL value was calculated based on the Log-Logistic model. The BMD_10_ (BMDL_10_) is the BMD (BMDL) corresponding to a 10 % additional risk

The results for females are presented in Table 3. At a BMR of 10 %, the BMDLs of UCd for Uβ_2_-MG were 1.59, 1.00, 1.94, 1.37, and 1.33 μg/g cr in females from Gansu, Hubei, Guizhou, Guangdong and Yunnan, respectively. The BMDL of UCd for Uβ_2_-MG in females from Guizhou was obviously higher than that of the other areas. The BMDLs of UCd for Uβ_2_-MG were lower in females than in males, with the exception of Yunnan.

### The BMD of UCd for the renal dysfunction index in the total population of all five provinces

Despite the fact that the mean UCd and Uβ_2_-MG levels among the subjects of some study areas were different, an obvious dose–response relationship existed between the UCd and the prevalence of renal dysfunction in both the study population of single area and the total study population of all five areas. Based on the Benchmark Dose Technical Guidance, datasets that are statistically and biologically compatible may be combined, resulting in increased statistical and biological confidence in the calculated BMD. Therefore, the overall BMD of UCd for Uβ_2_-MG in the total population was then estimated by combining the five data sets from all 6103 participants. As shown in Table 3, the overall BMDLs of UCd for Uβ_2_-MG with BMRs at 10 % were 2.00 μg/g cr in males and 1.69 μg/g cr in females. The BMDLs in the total population of females were slightly lower than those in males (Table 3).

## Discussion

The BMD of UCd for renal dysfunction in the population of five different Chinese provinces was assessed individually using Uβ_2_-MG as the effect indicator, and the results showed that the estimated BMD value was significantly affected by the participants’ geographic region and gender. For the first time, the BMD of UCd for kidney dysfunction in the Chinese population was further evaluated by combining the five data sets from all 6103 subjects. The overall BMDLs of UCd for Uβ_2_-MG with an excess risk of 10 % was 2.00 μg/g cr in males and 1.69 μg/g cr in females of the total sample population. These values were markedly lower than the reference level of 5 μg/g cr for Cd-related kidney effects recommended by the World Health Organization (WHO) [29].

Reports on individuals living in Cd-polluted and non-Cd-polluted areas have shown a close relationship between UCd excretion and the total body burden of Cd or lifetime Cd intake [13]. Therefore, UCd is considered to be a useful indicator of the internal dose of Cd exposure. Uβ_2_-MG was chosen as an indicator for Cd-induced renal effects [13, 14, 18]. The Uβ_2_-MG level of 1000 μg/g cr, which is from GB/T 17221–1998 in China, was used as the cut-off value. The threshold level for Uβ_2_-MG that is associated with the conversion from reversible to irreversible renal damage was determined to be 1000 μg/g cr of Uβ_2_-MG excretion [32]. Kobayshi et al. employed 84 % and 95 % of the upper limit values of Uβ_2_-MG of the non-smoking population as the cut-off values [11]. Usually, the 95 % upper limit values, which were 994 μg/g cr for men and 784 μg/g cr for women in their study, were used. When 95 % of the upper limit value or 1000 μg/g cr were used as a cut-off value, the value was the level over which the renal damage may be irreversible [13].

It is difficult to arrive at a conclusion on the relationship between the mean UCd and Uβ_2_-MG levels and the corresponding prevalence of the elevated UCd and Uβ_2_-MG levels by geographic regions. In some study areas, e.g., Hubei and Guangdong, the high UCd levels in the sample population corresponded well with the high Uβ_2_-MG levels as well as the high prevalence of the elevated UCd and Uβ_2_-MG levels (Table 1). Notably, the results observed in some other areas are different. The UCd level and the corresponding prevalence of elevated UCd levels in the subjects from Guizhou, which had more serious environmental [7] and food (e.g., rice) Cd pollution (detailed information will be reported in another study), were significantly higher than that observed in the other study areas (Table 1). However, the Uβ_2_-MG levels of the subjects in the Guizhou province were the lowest, despite their high Cd exposure levels. The corresponding prevalence of the elevated Uβ_2_-MG levels was also lower than that observed in most other study areas. Previous studies also showed that the Uβ_2_-MG levels of a sample population in a slightly exposed area were even higher than that observed in another highly exposed area (699 vs. 507 μg/g cr), whereas the UCd levels of the subjects in the former were significantly lower (1.4 vs. 7.9 μg/g cr) [33]. These results may be due to large differences of the ethnic group and lifestyle of the study population from different geographic regions. Even so, the Chi-squared linear trend test (*p* < 0.05) demonstrated the positive correlation between the prevalence of renal dysfunction and the UCd levels in the sample population from each geographic region. A positive correlation has also been reported in some previous studies of the Chinese and Japanese populations [34, 35], which indicates that Uβ_2_-MG may be an ideal biomarker for Cd-induced renal effects.

Thus far, the critical UCd levels for kidney effects have displayed large variations. A study based on the Swedish population showed that the BMDLs of UCd related to renal dysfunction ranged from 0.5 μg/g cr to 1.2 μg/g cr [17]. The BMDLs observed in Japanese samples varied from 1.4 μg/g cr to 11.4 μg/g cr [13–16]. The BMDLs of 2.13–4.85 μg/g cr were obtained based on two sets of data from occupational epidemiology in China [19]. The BMDLs of UCd for the renal effects were between 0.44 μg/g cr and 2.62 μg/g cr in another Chinese population from Cd-exposed areas [21]. These findings show that the BMDLs for the Cd-induced adverse health effects may be affected by many factors, such as race, effect biomarkers, sampling differences and BMD analysis methods. In the current study, the higher BMDLs of UCd for Uβ_2_-MG were observed in both males and females from the Guizhou province compared to the other provinces (Table 3). The selection of the sample populations affected the final BMDL values when a similar number of subjects from each province and the same race, effect biomarker, sampling and analytical procedures and BMD methods were considered. The present results showed that the geographic region selection significantly affected the BMDL evaluation. Because there was little difference between the prevalence of Uβ_2_-MG in the males and females of the Hubei (38.8 %–40.3 %) and Guangdong (40.3 %–41.3 %) provinces, the BMD values were quite similar (1.00 to 1.13 μg/g cr vs. 1.10 to 1.33 μg/g cr). The frequency that exceeded the standard of Uβ_2_-MG in the sample populations of Gansu, Guizhou and Guangdong ranged from 19.5 % to 31.6 %, which were significantly lower than 38.8 % to 42.7 % in the other two provinces (Table 1). The corresponding BMDLs were higher than those observed in the Hubei and Guangdong (1.37 to 3.66 μg/g cr vs. 1.00 to 1.33 μg/g cr). Previous studies reported that the prevalence of Uβ_2_-MG in females from Cd-polluted areas [13] and non-polluted areas [30] was 30.4 % and 12.9 %, respectively, and the corresponding BMD values of UCd were also quite different (1.5 μg/g cr vs. 3.3 μg/g cr) [13, 30]. Notably, the slightly exposed region (Hubei) showed the lowest BMDs for both males and females, whereas the highest values were observed in Guizhou. These results may be partially due to significantly higher Cd-exposure levels in Guizhou. Subjects who are living in highly polluted areas may become somewhat more resistant to Cd toxicity than those in slightly polluted areas. In addition, large differences of the ethnic groups as well as different lifestyles may also affect Cd’s toxic effect. More than 30 % individuals of the total population in Guizhou belong to minority ethnic groups, while this value in Hubei is only approximately 4 % (i.e., most of local residents in Hubei are the Chinese Han nationality).

In addition, the BMDLs of UCd for renal dysfunction were related to gender in addition to the sample population. Previous findings on the effects of gender on BMD values were different. In some studies, the BMDLs for Cd-induced renal dysfunction in females were higher than those in males [13, 16, 36]. However, other studies reported that the BMDLs were lower in females [14, 15], which indicated that the female subjects appear to be somewhat more sensitive to Cd damage than the male subjects. In the present study, the BMDLs of UCd for Uβ_2_-MG in females were lower than those of the males from each province, with the exception of the Yunnan province (Table 3 and 4). The overall BMDLs of the total population from all five provinces were also slightly lower in females (Table 3). Therefore, these results provide additional support to the latter findings.

The data obtained from all 6103 subjects from the five study areas were used to evaluate the reference level of Cd-induced kidney effects. This was performed to increase the statistical and biological confidence in the calculated BMD. The present investigation on the BMD has a greater statistical power than many previous studies of this type, particularly those for Chinese populations. The number of subjects in the present study was as high as 6103, much larger than the number (*N* = 374–790) of Chinese subjects in previous studies [18–21]. The sample size was also relatively larger than the Japanese populations in the previous studies of Kobayashi et al. (*N* = 2778) [13], Suwazono et al. (*N* = 1270) [15] and Suwazono et al. (*N* = 3103) [16]. Although the number of subjects in a previous BMD study of the Japanese population reached 6032 [36], these subjects came from different surveys that were completed separately in 1982 and 1998–2000 [34, 36]. In the present study, all participants were in the same survey and the same time frame. Furthermore, the exposure range was relatively broader than that of the previous studies [13, 30]. As reported in a previous study based on the population in non-polluted areas, the frequency of subjects with UCd concentrations above 7 μg/g cr was approximately 8 % [13]. The frequency in another study based on the population in polluted areas was 19 % [30]. In the current study, the frequency of subjects with UCd concentrations above 6 μg/g cr reached 40 %, based on the survey in the polluted provinces, which indicated much broader Cd-exposure levels in these subjects. In the present study, the estimated BMDL of UCd for Uβ_2_-MG with BMRs set at 10 % was 2.00 μg/g cr in males and 1.69 μg/g cr in females. The BMDLs obtained in the present study were significantly lower than other reports on the BMDLs of 3.99–12.18 μg/g cr in the Chinese population [18] and 2.4–11.4 μg/g cr in the Japanese population [13, 16, 32]. The present results were similar to those obtained in Swedish females (0.5–1.2 μg/g cr) [17], the Japanese population (1.4–2.6 μg/g cr) [15], the Chinese population that was also exposed to arsenic (0.9–1.2 μg/g cr) (Hong et al., [37]), and another Chinese population (0.44–0.53 μg/g cr) [21].

Previous studies have reported that the mortality risk significantly increases among the subjects with UCd values higher than 3 μg/g cr after age adjustment [38]. Cd toxicity has already been evaluated by several international bodies, including the International Agency for Research on Cancer, the Joint Food and Agriculture Organization (FAO)/WHO Expert Committee on Food Additives (JECFA) (2003, 2006, 2005, 2010) [39, 40], the European Commission [41], and the Agency for Toxic Substances and Disease Registry [42]. In 2010, the 73rd JECFA established a provisional tolerable monthly intake (PTMI) of 25 μg/month/kg body weight (bw) based on the findings of previous epidemiological studies [40]. The European Food Safety Authority recommended a considerably lower tolerable weekly intake of 2.5 μg/week/kg bw (i.e., nearly 10.7 μg/month/kg bw) based on an analysis of the relationship between UCd levels and tubular proteinuria [43]. The Scientific Committee on Toxicity, Ecotoxicity and the Environment of EC reported that adverse health effects may occur even at a lower level of 0.5 μg/g cr [44]. Recently, several guidance values for Cd exposure have become available, such as bio-monitoring equivalents (2 μg/g cr) [45] and human bio-monitoring values (1.0 μg/L for adults; 0.5 μg/L for children/adolescents) [46]. These values were significantly lower than the reference level of 5.0 μg/g cr for Cd-induced kidney dysfunction recommended by the WHO [29] and MOHC [47]. In the present study, the estimated threshold values for Cd-induced renal dysfunction were within the range of 1.00 μg/g cr to 3.66 μg/g cr, which provides additional scientific support for identifying a lower biological limit for Cd exposure and a provisional tolerable monthly intake (PTMI).

This study covers a wide range of ages and Cd exposure levels in both men and women. Furthermore, when considering the larger sample population size than that of previous studies [18–21], we believe that this study improved the accuracy of the BMD of UCd for renal dysfunction in the Chinese population. However, this study has limitations that should be considered in future studies. The results of Japanese studies showed that the prevalence of Uβ_2_-MG is increased with increasing age in both sexes. The obtained BMDL gradually decreased with increasing age, which indicated that the margin between the threshold level and average excretion level of urinary Cd was small in the older population in Japan. Therefore, it is important to evaluate the threshold level of Cd exposure in the general Chinese population, while accounting for an age effect to prevent Cd’s toxic effects. Furthermore, there are 56 ethnic groups from more than 30 provinces, municipalities and autonomous regions in China. Large differences in the ethnic group, lifestyle, and diet and health history of the study population may also be important factors related to Cd-induced adverse health effects. Further studies are needed to reach a conclusion.

## Conclusions

The BMD value of UCd for Uβ_2_-MG was related to the participants’ geographic region and gender. The effect of the participants’ geographic region on the BMD estimate may be partially attributable to the large differences in Cd exposure level, ethnic group, lifestyle and diet of the sample population in these study areas. The overall BMDLs of UCd for Uβ_2_-MG with an excess risk of 10 % in this large sample survey of 6103 subjects was be 2.00 μg/g cr in males and 1.69 μg/g cr in females, which were significantly lower than the reference level for Cd-related kidney dysfunction suggested by the WHO. These findings provide additional scientific support for reconsidering the threshold exposure value and PTMI for Cd to protect people from Cd-induced adverse health effects.

## Abbreviations

ATSDR: Agency for Toxic Substances and Disease Registry
BMD: Benchmark dose
BMDL: Benchmark dose low
BMR: benchmark response
EC: European Commission
EFSA: European Food Safety Authority
GM: Geometric mean
IARC: International Agency for Research on Cancer
JECFA: Joint FAO/WHO Expert Committee on Food Additives
LOAEL: Lowest observed adverse effect level
MOHC: Minister of Health of the People’s Republic of China
NOAEL: No observed adverse effect level
PTMI: Provisional tolerable weekly intake
TWI: Tolerable weekly intake
UCd: Urinary cadmium
Uβ_2_-MG: Urinary β_2_-microglobulin
WHO: World Health Organization
